# The a3 isoform of subunit a of the vacuolar ATPase localizes to the plasma membrane of invasive breast tumor cells and is overexpressed in human breast cancer

**DOI:** 10.18632/oncotarget.10063

**Published:** 2016-06-15

**Authors:** Kristina Cotter, Rachel Liberman, GeHong Sun-Wada, Yoh Wada, Dennis Sgroi, Stephen Naber, Dennis Brown, Sylvie Breton, Michael Forgac

**Affiliations:** ^1^ Department of Developmental, Molecular and Chemical Biology, Tufts University School of Medicine and The Program in Cellular and Molecular Physiology, Sackler School of Graduate Biomedical Sciences, Tufts University, Boston, Massachusetts 02111, USA; ^2^ Program in Membrane Biology/Nephrology Division, Center for Systems Biology, Massachusetts General Hospital/Harvard Medical School, Boston, Massachusetts, 02114, USA; ^3^ Department of Biochemistry, Faculty of Pharmaceutical Sciences, Doshisha Women's College, Kyotanabe, Kyoto 610-0395, Japan; ^4^ Division of Biological Science, Institute of Scientific and Industrial Research, Osaka University, Ibaraki, Osaka 567-0047, Japan; ^5^ Department of Pathology, Molecular Pathology Unit, Massachusetts General Hospital, Charlestown, Massachusetts 02129, USA; ^6^ Department of Pathology, Tufts Medical Center, Boston, Massachusetts 02111, USA

**Keywords:** vacuolar ATPase, proton transport, acidification, invasion, breast cancer

## Abstract

The vacuolar (H^+^)-ATPases (V-ATPases) are a family of ATP-driven proton pumps that acidify intracellular compartments and transport protons across the plasma membrane. Previous work has demonstrated that plasma membrane V-ATPases are important for breast cancer invasion *in vitro* and that the V-ATPase subunit a isoform a3 is upregulated in and critical for MDA-MB231 and MCF10CA1a breast cancer cell invasion. It has been proposed that subunit a3 is present on the plasma membrane of invasive breast cancer cells and is overexpressed in human breast cancer. To test this, we used an a3-specific antibody to assess localization in breast cancer cells. Subunit a3 localizes to the leading edge of migrating breast cancer cells, but not the plasma membrane of normal breast epithelial cells. Furthermore, invasive breast cancer cells express a3 throughout all intracellular compartments tested, including endosomes, the Golgi, and lysosomes. Moreover, subunit a3 knockdown in MB231 breast cancer cells reduces *in vitro* migration. This reduction is not enhanced upon addition of a V-ATPase inhibitor, suggesting that a3-containing V-ATPases are critical for breast cancer migration. Finally, we have tested a3 expression in human breast cancer tissue and mRNA prepared from normal and cancerous breast tissue. a3 mRNA was upregulated 2.5-47 fold in all breast tumor cDNA samples tested relative to normal tissue, with expression generally correlated to cancer stage. Furthermore, a3 protein expression was increased in invasive breast cancer tissue relative to noninvasive cancer and normal breast tissue. These studies suggest that subunit a3 plays an important role in invasive human breast cancer.

## INTRODUCTION

Cancer cells are able to survive and proliferate in tumor microenvironments that are more acidic than normal, maintaining a slightly alkaline intracellular pH despite the lower than normal extracellular pH [1]. This unique ability also affects the metastatic potential of cancer cells. Metastasis, or the spread of a primary tumor to secondary sites within the body, requires that a cancer cell be able to migrate and invade through extracellular matrix [2]. A more alkaline cytosolic pH can enhance cytoskeletal remodeling necessary for cell migration and an acidic microenvironment enhances cancer cell invasion by promoting the activity of pH-dependent proteases [1]. Thus, targeting the mechanisms by which cancer cells survive in acidic microenvironments represents a possible strategy for limiting cancer metastasis.

The V-ATPase is an ATP-dependent proton pump that is ubiquitously expressed in eukaryotic cells [3–6]. The primary role of the V-ATPase is to transport protons into intracellular compartments or the extracellular space. The V-ATPase consists of two functional domains. The cytoplasmic V_1_ domain is responsible for ATP hydrolysis and contains eight different subunits (A-H) while the membrane-embedded V_0_ domain is responsible for proton transport and contains five different subunits (a, c, c’, d, and e) [3]. Within the cell, the V-ATPase is present in endosomes, lysosomes, the Golgi, and secretory vesicles, where it is involved in such functions as receptor-mediated endocytosis, protein trafficking, zymogen activation, and the transport of small molecules. V-ATPases are also found on the plasma membranes of specialized acid secreting cells. These include osteoclasts, where V-ATPases are involved in bone resorption, renal intercalated cells, where they function in pH homeostasis by secreting acid from the blood into the urine, and epididymal clear cells, where they are involved in sperm maturation [3–7].

Localization of the V-ATPase to specific membranes within the cell is controlled by the 100-kDa subunit a of the V_0_ domain [8, 9]. Subunit a is present in four isoforms in mammals (a1-a4) [10]. The a1 and a2 isoforms have been identified within intracellular compartments, with a1 present in synaptic vesicles [11] while a2 localizes to the Golgi in pre-osteoclast cells [12] and to apical endosomes in renal proximal tubular cells [13]. Subunits a3 and a4 are present at the plasma membrane of specialized cells and, for a3, may also be present within cells. a3 is localized to the plasma membrane of osteoclasts and to lysosomes in pre-osteoclast cells [7, 14]. a3 has also been identified in insulin-containing secretory vesicles in pancreatic β cells [15]. The a4-containing V-ATPases localize to the apical plasma membrane of both renal intercalated cells and epididymal clear cells [16–19]. Mutations in a3 and a4, though not lethal, can lead to osteopetrosis and renal tubular acidosis, respectively [14, 17].

A number of recent studies have demonstrated that the V-ATPase plays critical roles in cancer cells, particularly with regard to invasion and migration. For example, the V-ATPase is expressed at the plasma membrane of invasive MB231 human breast cancer cells, but not in noninvasive MCF7 cells [20]. Furthermore, inhibition of the V-ATPase with the specific inhibitors bafilomycin and concanamycin A results in a significant reduction in the *in vitro* invasive and migratory capabilities of MB231 cells [20–22]. Plasma membrane V-ATPase expression and dependence of invasion and migration on V-ATPase activity has also been observed in other breast cancer cell lines as well as in other cancer cell types, including pancreatic, prostate, ovarian, and liver cancer as well as melanoma and Ewing sarcoma [23–32].

Isoforms of subunit a of the V-ATPase have been shown to play a critical role in cancer cell invasion. Subunits a3 and a4, which are known to localize the V-ATPase to the plasma membranes of specialized acid-secreting cells, are upregulated at the mRNA level in invasive MB231 breast cancer cells relative to noninvasive MCF7 cells [22]. Subunit a3 is also upregulated at the mRNA level in invasive MCF10CA1a breast cancer cells relative to the parental MCF10a breast epithelial cell line [23]. siRNA-mediated knockdown of a3 and a4 reduces MB231 cell invasion while knockdown of a3 also reduces MCF10CA1a invasion [22, 23]. Importantly, overexpression of subunit a3 in the parental MCF10a breast epithelial cell line enhances both invasiveness and plasma membrane V-ATPase expression [23]. Subunit a3 has also been shown to be upregulated in and critical for the invasion of melanoma cells [32]. Collectively, these results suggest that overexpression of subunit a isoforms, particularly a3, may increase trafficking of the V-ATPase to the plasma membrane, where it then contributes to cancer cell invasion. The contribution of the subunit a isoforms to breast cancer cell migration has not yet been assessed.

Because complete ablation of V-ATPase activity is lethal to mammalian cells [33–35], it is of interest to identify particular populations of V-ATPase that contribute to tumor cell invasion in order to develop safe and specific inhibitors of cancer metastasis. We have recently shown that specific ablation of plasma membrane V-ATPases inhibits *in vitro* invasion and migration of MB231 cells [21]. While, as noted above, a3 has been implicated in plasma membrane targeting of V-ATPases and *in vitro* invasion of a number of cancer cell lines, it is not known whether a3 is actually present in V-ATPase complexes present at the surface of tumor cells. This is important since it is possible that a3-containing V-ATPases function instead within intracellular compartments of tumor cells to aid in the delivery of V-ATPases to the cell surface. Furthermore, the expression of subunit a3 in human breast cancer samples has not yet been assessed. It is thus of critical importance to gain a better understanding of the expression and function of subunit a3 in breast cancer in order to evaluate a3-containing V-ATPases as a potential therapeutic target for the treatment of breast cancer.

To more directly assess the localization, function, and expression of subunit a3 in human breast cancer, we have employed an antibody that is specific for this isoform. Immunofluorescence studies indicate that subunit a3 localizes to the leading edge of several highly invasive human breast cancer cell lines, but is not present at the plasma membrane of noninvasive MCF10a breast epithelial cells. Interestingly, expression of a3 in the invasive cells is not confined to the plasma membrane, but is also present in endosomes, Golgi and, in particular, lysosomes. To complement previous studies of the role of a3 in tumor cell invasion, we now show that knockdown of a3 also inhibits migration of MB231 breast cancer cells. We have also found, using qRT-PCR, that a3 mRNA is overexpressed in samples of human breast tumors relative to normal breast tissue. Finally, using immunofluorescence, we have shown that a3 is present at high levels in invasive human breast carcinoma relative to noninvasive breast tumors and normal breast tissue. These results suggest that subunit a3 plays a critical role in at least some human breast cancers and may represent a novel therapeutic target to inhibit breast cancer metastasis.

## RESULTS

### Subunit a3 localizes to the plasma membrane and the leading edge of invasive breast cancer cells

Despite the evidence suggesting its importance in breast tumor cell invasion discussed above, the localization of the a3 isoform the V-ATPase subunit a in breast cancer cells is not known. In order to assess the localization of a3, a chicken monoclonal antibody specific for the mouse a3 was employed. This antibody was previously shown to be specific for the a3 isoform of the mouse V-ATPase and has been used previously to assess a3 expression in mouse melanoma cells [32, 36]. The mouse and human subunit a isoform sequences exhibit a high degree of homology (Supplementary Figure S1A). Mouse and human subunit a3 protein sequences are 84% identical [37–39]. The a3 antibody was raised against amino acids 658-720 of the mouse a3 sequence, corresponding to amino acids 657-716 in the human sequence (Supplementary Figure S1B) [36]. This region is unique as compared to the a1, a2, and a4 human protein sequences (Supplementary Figure S1C) [36–39]. Moreover, the anti-a3 antibody recognizes a band of the expected molecular mass (∼120-kDa) by Western blot of whole cell lysates from human breast cells (see below), indicating that this antibody recognizes the human a3 protein.

**Figure 1:**
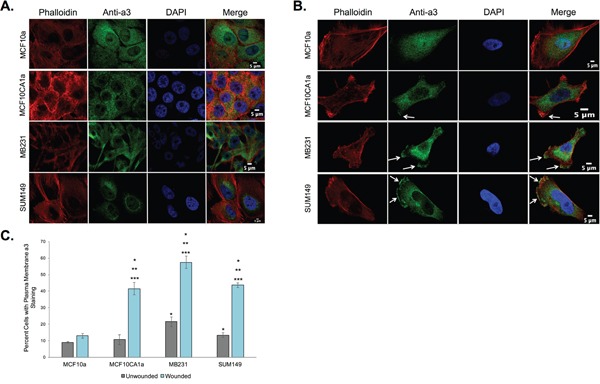
Subunit a3 localizes to the plasma membrane of human breast cancer cells MCF10a, MCF10CA1a, MB231, and SUM149 cells were grown to confluence on poly-d-lysine coated coverslips. To assess the effects of cell migration on a3 localization, a wound was made in the cell monolayer and cells were allowed to migrate for 4 h. Cells were then fixed, permeabilized, and immunostained using antibodies against subunit a3 of the V-ATPase and Alexa Fluor® 568 phalloidin to stain actin followed by incubation with secondary antibodies as described under Materials and Methods. Images were taken with identical exposure times and antibody concentrations. **A.** Shown are representative images of confluent MCF10a, MCF10CA1a, MB231, and SUM149 cells stained for phalloidin (*left*), subunit a3 *(second from left*), DAPI (*second from right*), and the merged images (*right*). **B.** Shown are representative images of MCF10a, MCF10CA1a, MB231, and SUM149 cells (with monolayer wounded) stained for phalloidin (*left*), subunit a3 *(second from left*), DAPI (*second from right*), and the merged images (*right*). *White arrows* indicate plasma membrane subunit a3. **C.** Quantification of plasma membrane staining for subunit a3 in confluent or migrating cells. To quantitate plasma membrane staining, 50-80 cells from three separate experiments were counted and the number of cells showing plasma membrane a3 localization was determined. The graphed data represents the percentage of cells showing a3 localization to the plasma membrane. All *error bars* indicate S.E. *, p < 0.05 relative to unwounded MCF10a cells. **, p < 0.05 relative to wounded MCF10a cells. ***, p < 0.05 relative to the unwounded cell of the same cell type.

Previous studies that have shown V-ATPase expression on the plasma membrane of highly invasive and migratory breast cancer cells [20–23] assessed V-ATPase localization by staining for subunits of the cytoplasmic V_1_ domain, and thus the localization of the a3 isoform of the V-ATPase remains unclear. To address this, immunocytochemistry was performed on a panel of breast cancer cells to measure a3 expression at the plasma membrane. Experiments were performed on both confluent monolayers and monolayers that had been scratched to induce cell migration, as described in Materials and Methods. As shown in Figure 1A, the breast epithelial cell line MCF10a and the invasive breast cancer cell line MCF10CA1a express little or no a3 at the plasma membrane when the cells are present in a monolayer. By contrast, when a leading edge is induced by wounding the monolayer, MCF10CA1a, MB231, and SUM149 cells all display a significant re-localization of a3 to the plasma membrane, including the leading edge (Figure 1B). This effect is not observed in the noncancerous MCF10a cells. These results are quantified in Figure 1C. It is of note that a small proportion of invasive MB231 and SUM149 breast cancer cells exhibit a3 at or near the plasma membrane when present in a monolayer (Figure 1C, not pictured in Figure 1A), likely due to the highly migratory nature of these cell lines. Overall, these data demonstrate for the first time that a3 is present at the plasma membrane of invasive and migratory breast cancer cells, suggesting that the plasma membrane V-ATPases previously shown to be critical for breast cancer cell migration and invasion contain the a3 isoform.

**Figure 2:**
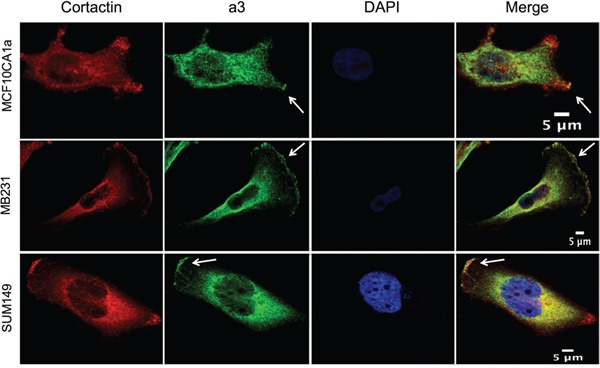
Subunit a3 colocalizes with a leading edge marker in migrating breast cancer cells MCF10CA1a, MB231, and SUM149 breast cancer cells were grown to confluence on poly-d-lysine coated coverslips. A wound was made in the cell monolayer and cells were allowed to migrate for 4 h in order to induce the formation of a leading edge. Cells were then fixed, permeabilized, and immunostained using antibodies against subunit a3 of the V-ATPase and the leading edge marker cortactin followed by incubation with secondary antibodies as described under Materials and Methods. Images were taken with identical exposure times and antibody concentrations. Shown are representative images of MCF10CA1a, MB231, and SUM149 cells stained for cortactin (*left*), a3 (*second from left*), and DAPI (*second from right*). The merged images are present to the far right. *White arrows* indicate subunit a3 and cortactin colocalization at the leading edge. The images depict representative staining from a minimum of two separate experiments, with 50-80 cells imaged per experiment for each condition tested.

As demonstrated in Figure 1B, subunit a3 localizes to regions of the plasma membrane of invasive and migratory breast cancer cells that appear to correspond to the leading edge. To confirm this, immunofluorescence was performed on migrating MCF10CA1a, MB231, and SUM149 cells using antibodies against a3 and cortactin, which is present at the leading edge of migrating cells [40]. As shown in Figure 2, colocalization of cortactin and a3 is observed in each of the breast cancer cell lines examined.

### Subunit a3 is involved in breast cancer cell migration

**Figure 3:**
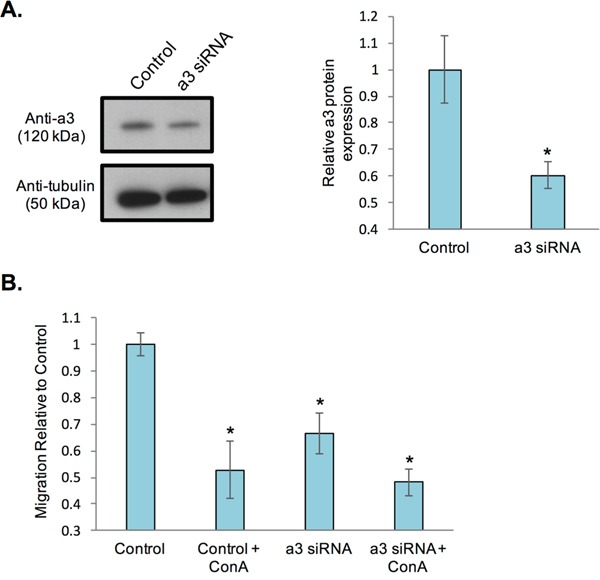
siRNA-mediated knockdown of subunit a3 in breast cancer cells reduces *in vitro* migration **A.** MB231 cells were incubated with an siRNA pool specific for subunit a3 or scrambled siRNA (‘control’). After 72 hours, cells were lysed and protein lysates separated by SDS-PAGE on 4-15% gradient acrylamide gels followed by transfer to nitrocellulose. Immunoblotting was performed using monoclonal antibodies against subunit a3 and tubulin. The blot displayed is representative from three separate siRNA experiments. Western blots from each experiment were quantitated and the ratio of subunit a3 expression in control versus a3 siRNA-transfected cells was calculated. The graphed data represent the average degree of a3 knockdown relative to control cells. *, p < 0.05 relative to control cells. **B.** Control and a3 siRNA-transfected cells were treated in the presence or absence of 100 μM of the V-ATPase specific inhibitor concanamycin A, plated onto FluorBlok™ inserts and allowed to migrate towards a chemoattractant on the *trans*-side of the well for an average of 20 hours. Cells were then stained with Calcein AM and the number of cells that had migrated to the *trans*-side were counted, with 3 wells analyzed per sample and an average of 10 images analyzed per well. Values are the mean of three independent siRNA experiments and expressed relative to control untreated cells. All *error bars* indicate S.E. *, p < 0.05 relative to control untreated cells.

The presence of subunit a3 at the leading edge suggests that it may play an important role in cancer cell migration and invasion. Indeed, previous work has found that loss of a3 in invasive MB231 and MCF10CA1a cells reduces *in vitro* invasiveness [22, 23]. While plasma membrane V-ATPases have been shown to be involved in *in vitro* breast cancer cell migration [21], the role of the subunit a isoforms in this process has not yet been assessed. To test whether a3 contributes to breast cancer cell migration, MB231 cells were transfected with a pool of siRNAs specific for a3, as has been previously described [22, 23], prior to measuring *in vitro* migration. a3 expression after siRNA transfection was assessed by Western blot of whole cell lysates. As shown in Figure 3A, transfection with a3-specific siRNAs significantly reduced a3 protein expression relative to control cells transfected with a scrambled siRNA pool. To determine the effect of partial knockdown of a3 on *in vitro* migration, a transwell migration assay was performed as described under Materials and Methods. As shown in Figure 3B, MB231 cells transfected with scrambled, non-targeting siRNAs show an approximately 50% reduction in migration upon treatment with the specific V-ATPase inhibitor concanamycin A, consistent with previous reports [20, 21]. Partial knockdown of subunit a3 reduced the migration of MB231 cells to a similar degree as observed with concanamycin A. Moreover, cells treated with a3-specific siRNAs together with concanamycin A showed migration that was not statistically different from cells subjected to either treatment alone. It is important to note that neither a3 knockdown nor a3 knockdown in combination with 24 h concanamycin A treatment impairs viability of MB231 cells relative to untreated controls (data not shown), and thus the reduction in migratory capability is not a result of cell death. Overall, these results demonstrate for the first time that a3-containing V-ATPases are the primary population of V-ATPases involved in MB231 cell migration. Together with previous studies demonstrating the role of plasma membrane and a3-containing V-ATPases in breast cancer cell invasion [21–23], these results suggest that a3-containing V-ATPases present at the plasma membrane represent a potential anti-metastatic target for treatment of breast cancer.

### Intracellular localization of a3 in breast cancer cells

**Figure 4:**
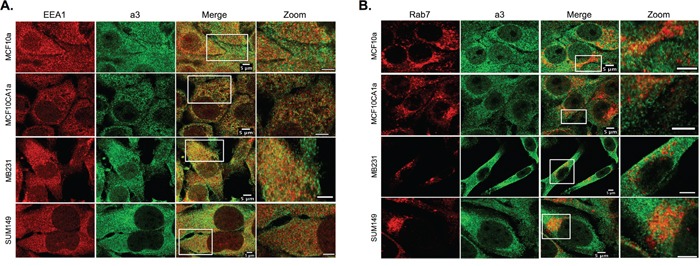
Intracellular localization of subunit a3 and markers for early and late endosomes in breast cancer cells MCF10a, MCF10CA1a, MB231, and SUM149 cells were grown on poly-d-lysine coated coverslips. To assess whether a3 localizes to endosomal compartments, cells were fixed, permeabilized, and immunostained using antibodies against subunit a3 of the V-ATPase and EEA1 or Rab7 as markers for early endosomes and late endosomes, respectively. Cells were then incubated with secondary antibodies as described under Materials and Methods. Images were taken with identical exposure times and antibody concentrations. **A.** Representative images of each of the cell lines stained for the early endosome marker EEA1 (*left*), subunit a3 (*second from left*), the merged images (*second from right*), and the inset of the merged image magnified 3.5X (*labeled ‘Zoom’, far right*). **B.** Representative images of each of the cell lines stained for the late endosome marker Rab7 (*left*), subunit a3 (*second from left*), the merged images (*second from right*), and the merged image magnified 3.5X (*labeled ‘Zoom’, far right*). Colocalization is indicated by the presence of yellow pixels. The images in *A* and *B* depict representative staining from a minimum of two separate experiments, with an average of 50 cells imaged per experiment for each condition tested.

**Figure 5:**
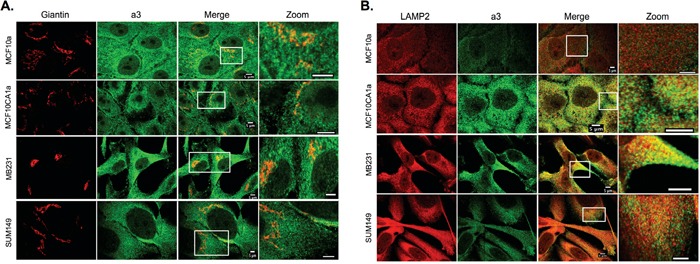
Intracellular localization of subunit a3 and markers for the Golgi apparatus and lysosomes in breast cancer cells MCF10a, MCF10CA1a, MB231, and SUM149 cells were grown on poly-d-lysine coated coverslips. To assess whether a3 localizes to the Golgi and lysosomes, cells were fixed, permeabilized, and immunostained using antibodies against subunit a3 of the V-ATPase and Giantin or LAMP2 as markers for the Golgi and lysosomes, respectively. Cells were then incubated with secondary antibodies as described under Materials and Methods. Images were taken with identical exposure times and antibody concentrations. **A.** Representative images of each of the cell lines stained for the Golgi apparatus marker Giantin (*left*), subunit a3 (*second from left*), the merged images (*second from right*), and the merged image magnified 3.5X (*labeled ‘Zoom’, far right*). **B.** Representative images of each of the cell lines stained for the lysosomal marker LAMP2 (*left*), subunit a3 (*second from left*), the merged images (*second from right*), and the inset of the merged image magnified 3.5X (*labeled ‘Zoom’, far right*). Colocalization is indicated by the presence of yellow pixels. The images in *A* and *B* depict representative staining from a minimum of two separate experiments, with an average of 50 cells imaged per experiment for each condition tested.

As shown in Figure 1A and 1B, intracellular staining for a3 is present in each of the breast cell lines tested. To determine the intracellular localization of a3, immunocytochemistry was performed on on MCF10a, MCF10CA1a, MB231, and SUM149 cells using both the anti-a3 antibody together with markers for various intracellular compartments. The markers employed are EEA1 (early endosomes), Rab7 (late endosomes), Giantin (Golgi) and LAMP2 (lysosomes (see Figures 4 and 5). The strongest colocalization observed was a3 with LAMP2, particularly in MB231 and MCF10CA1a cells (Figure 5B), suggesting the presence of a significant number of a3-containing V-ATPases in lysosomes of invasive breast cancer cells. Interestingly, relatively little colocalization of a3 and LAMP2 was observed in the non-tumorigenic MCF10a cells. Significant co-localization of a3 was also observed with EEA1 (Figure 4A) and Giantin (Figure 5A), suggesting the presence of a3-containing V-ATPases in early endosomes and the Golgi as well as lysosomes. By contrast, there was generally less colocalization of a3 and Rab7 (Figure 4B), suggesting that a3-containing V-ATPases may be less abundant in late endosomes. Nevertheless, the results demonstrate that a3-containing V-ATPases are not exclusively localized to a single compartment in any of the cell lines examined.

### a3 is overexpressed in invasive human breast cancer tissues relative to noninvasive and normal breast tissue

**Figure 6:**
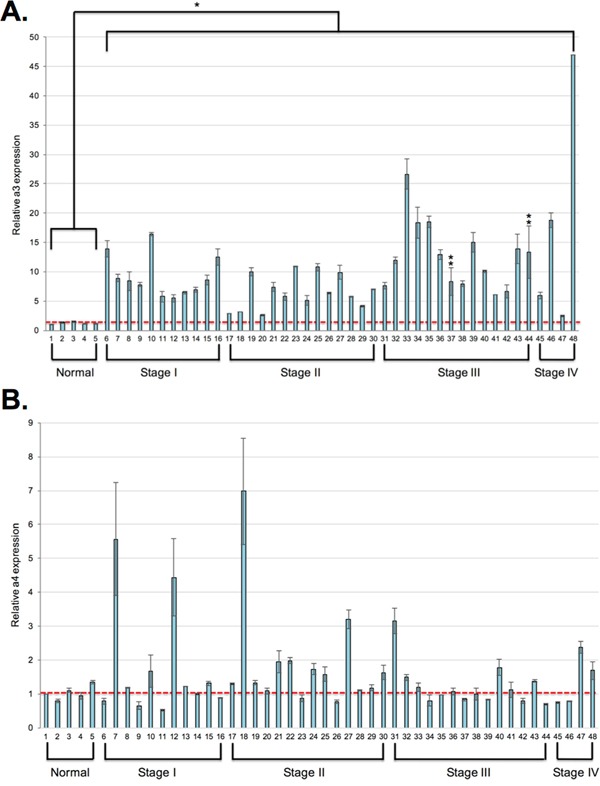
Subunit a3 mRNA is overexpressed in human breast cancer samples relative to normal tissue qPCR using a3-specific primers **A.** or a4-specific primers **B.** was performed on the Origene breast cancer cDNA array II as described under Materials and Methods. Data was analyzed using the ΔΔC_t_ method. Values represent a3 or a4 expression levels relative to the first normal breast tissue sample (sample 1, indicated by the red dotted line) and are the mean of two separate experiments. *Error bars* indicate S.E. In the a3 graph, *, p < 0.05 relative to the normal samples and **, p < 0.11 relative to the normal samples.

Expression of a3 in human breast cancer samples has not yet been determined. It is critical to assess a3 expression in human breast cancers in order to evaluate a3 as a potential anti-metastatic target *in vivo*. To measure a3 mRNA expression in human breast cancer samples, qRT-PCR was performed using a3-specific primers on a breast cancer cDNA panel (Origene). The array contained 5 normal breast tissue samples and 43 breast cancer samples of obtained from tumors of various clinical stages. Information concerning age and hormone receptor status was provided for each sample. As shown in Figure 6A, a3 mRNA expression was upregulated in all of the breast cancer samples by 2.5 to 47-fold relative to the control samples. The degree of overexpression did not correlate with patient age or hormone receptor status (not shown). Interestingly, the highest degree of overexpression was generally observed in samples from tumors of stage III and IV. Though this may be indicative of an important role for a3 in the invasiveness of at least some breast tumor cells, it should be noted that a3 is present at elevated levels in all breast tumor samples, even the noninvasive tumors. These results suggest a3 likely functions in roles other than tumor cell migration and invasion. Because of the large amount of metabolic acid generated by tumor cells as a result of their reliance on glycolysis, overexpression of a3 in tumor cells may direct more V-ATPase to the plasma membrane, where it can aid in ridding the cell of this excess acid.

As we currently lack specific antibodies that reliably recognize the other human subunit a isoforms, we were unable to examine their localization and protein expression in breast cancer samples. However, as previous work in our laboratory has found that a4 is overexpressed at the mRNA level in invasive MB231 breast cancer cells relative to noninvasive MCF7 cells, and that it is critical for the *in vitro* invasiveness of MB231 cells, we performed qRT-PCR on the breast cancer cDNA panel using a4-specific primers. As shown in Figure 6B, a4 expression was enhanced in only a small fraction of the breast cancer samples relative to normal tissue. Furthermore, the increase in expression was moderate relative to what was observed for subunit a3.

**Figure 7:**
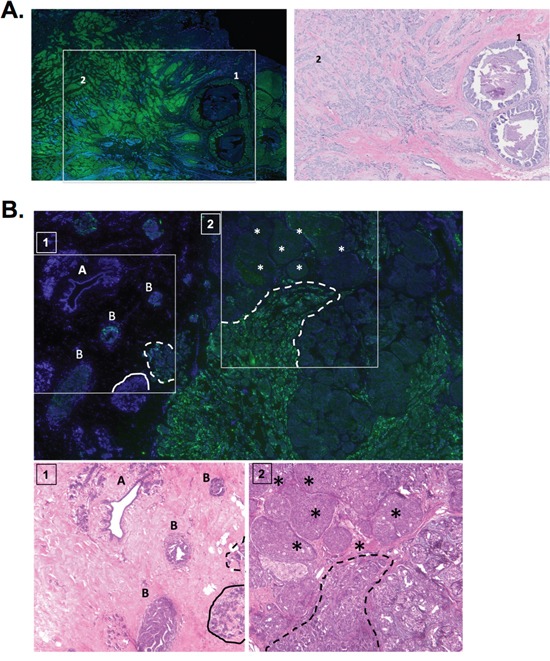
Subunit a3 protein is overexpressed in invasive breast cancer tissue relative to noninvasive breast cancer and normal tissue **A.** Immunohistochemistry for subunit a3 was performed as described under Materials and Methods on 5 breast cancer tissue samples. Shown on left is a representative image depicting a3 expression (green) and DAPI (blue) in a tissue sample containing ductal carcinoma *in situ* (labeled ‘1’) and invasive breast carcinoma (labeled ‘2’). H&E staining for the inset can be seen to the right. The sample from the representative image is classified as invasive ductal carcinoma, 2.1 cm (T2), grade 2 tumor with negative lymph nodes (N0). Tumor was ER^+^, PR^+^, and HER2^−^. **B.** Immunohistochemistry for subunit a3 was performed as described under Materials and Methods on 5 human samples that contained breast cancer adjacent to normal tissue. Shown is a representative image depicting a3 expression (green) and DAPI (blue) in normal breast ducts and lobules (labeled ‘A’), hyperplastic tissue (labeled ‘B’), benign breast lobule (indicated by solid line), solid tumor (labeled by asterisks), and invasive carcinoma (indicated by dotted line). H&E staining for the insets 1 & 2 can be seen below. The sample from the representative image is classified as invasive ductal carcinoma, 2.1 cm (T2), grade 3.5 with 13 lymph nodes with metastases (N2). Tumor was ER^+^, PR^+^, and HER2^.^ Immunohistochemical images were interpreted independently by pathologists at Massachusetts General Hospital (Dr. Dennis Sgroi) and Tufts Medical Center (Dr. Stephen Naber).

The results from the breast cancer cDNA panel suggest that a3 is significantly overexpressed in breast cancer tissue relative to normal breast tissue. To further examine this, immunohistochemistry was performed on human breast cancer tissue samples using the a3-specific antibody described above. First, we examined breast cancer tissue samples that contain invasive breast carcinoma tissue adjacent to noninvasive breast tumor tissue. The representative image in Figure 7A depicts a breast tumor tissue sample that contains ductal carcinoma *in situ* (DCIS, labeled ‘1’), which is a type of noninvasive breast cancer that originates from inside the milk duct. DCIS is considered noninvasive as it has not yet spread beyond the basement membrane of the duct into adjacent breast tissue [41]. The tissue sample in Figure 7A also contains invasive breast carcinoma cells adjacent to the DCIS tissue (labeled ‘2’). Interestingly, the invasive breast tissue in Figure 7A exhibits greater staining for a3 relative to the DCIS tissue, suggesting that a3 expression is greater in invasive breast cancer cells *in vivo*.

Next, immunohistochemistry was performed on human breast tumor tissue samples that contain normal breast tissue adjacent to breast tumor tissue. The representative image in Figure 7B depicts a breast tumor tissue sample containing normal breast ducts and lobules (indicated by the solid line and the letter ‘A’) and hyperplastic tissue (indicated by the letter ‘B’), which represents highly proliferative (though not necessarily precancerous) tissue. Adjacent to the normal tissue is noninvasive solid tumor tissue, or a breast tumor containing cells that have not yet invaded the surrounding breast tissue (indicated by the asterisks) as well as invasive breast carcinoma (indicated by the dotted line). Interestingly, staining for a3 is greatest in invasive breast carcinoma as compared to the noninvasive and hyperplastic breast tissue. Furthermore, normal breast tissue did not stain for a3. Overall, the results described in Figure 7 suggest that a3 is overexpressed in human breast cancer and that its expression increases with invasive potential.

## DISCUSSION

Although several studies have examined the role of the a3 isoform of the V-ATPase subunit a in breast cancer cell invasion *in vitro* [22–23], much remains unknown with regard to the localization, function, and expression of a3 in human breast cancer. It has been demonstrated that a3 mRNA is upregulated in several invasive breast cancer cell lines and that its knockdown reduces breast cancer cell invasion *in vitro* [22, 23]. Furthermore, overexpression of a3 in the MCF10a breast epithelial line enhances invasiveness and plasma membrane V-ATPase localization [23]. It has also recently been shown that plasma membrane V-ATPases are critical for the invasion and migration of MB231 breast cancer cells [21]. As previous studies did not examine the localization of subunit a3 in breast cancer cells, it was unclear whether a3-containing V-ATPases are actually present at the plasma membrane of breast cancer cells or were merely important in the trafficking of V-ATPases to the cell surface. Furthermore, the expression of a3 in human breast tumors had not been assessed. The present study aimed to determine both the localization and expression of the a3 isoform in human breast cancer samples in order to gain insight into its suitability as a target to limit breast cancer metastasis.

In this study, we have demonstrated that a3-containing V-ATPases are present intracellularly in normal MCF10a breast epithelial cells as well as several invasive breast cancer cell lines. Importantly, when the breast cancer cells are induced to migrate, a3 strongly localizes to the leading edge. Subunit a3 has previously been shown to be critical for MB231 and MCF10CA1a breast cancer cell invasion [22, 23]. Results from the present study indicate for the first time that a3 is the predominant subunit a isoform contributing to breast cancer cell migration. This, in combination with our previous work demonstrating a role for plasma membrane V-ATPases in breast cancer cell invasion and migration [21], highlight the importance of a3-containing, plasma membrane V-ATPases in these processes.

Our colocalization studies in normal MCF10a breast epithelial cells and the three invasive breast cancer cell lines provide insight into intracellular functions of a3-containing V-ATPases. Our results indicate that a3 at least partially colocalizes with markers for early endosomes and the Golgi apparatus in all breast cell lines tested, suggesting that a3-containing V-ATPases may be involved in endocytosis, protein trafficking or protein modification in both normal and breast tumor cells. Interestingly our results indicate that in each of the invasive breast cancer cell lines, a3 strongly localizes to lysosomes, suggesting a role in protein degradation. This was not observed in the non-tumorigenic MCF10a cell line, indicating that the intracellular localization patterns of a3 may be altered in cancer. Lysosomes undergo a number of changes during malignant transformation, including increased protease activity and trafficking towards the leading edge of migrating cells [42–44]. Both of these properties would promote the release and activation of proteases into the extracellular space that could enhance the invasiveness of breast tumor cells [43]. Strong colocalization of a3 with lysosomes in breast cancer cells suggests that a3-containing V-ATPases are likely involved in these processes.

While the mechanisms involved in delivery of a3 to the leading edge of breast cancer cells is unknown, it is possible that this relocalization is similar to that which occurs in osteoclasts upon maturation. Mature osteoclasts, or bone resorbing cells, are one of the few specialized cell types in the body that normally express plasma membrane V-ATPases. Expression of a3-containing V-ATPases at the osteoclast ruffled border membrane is critical for proper bone resorption [6]. Previous studies of osteoclast progenitor cells have demonstrated that a3 localizes to late endosomes and lysosomes, and that upon differentiation into mature osteoclasts, it relocalizes to the plasma membrane, possibly through fusion of secretory lysosomes with the cell surface [7]. Moreover, a change in interaction between the V-ATPase and actin filaments has been implicated in this process [45]. The strong colocalization of a3 with the lysosomal marker in breast cancer cells, but not the normal breast cell line, suggests the possibility that plasma membrane V-ATPases containing a3 may be derived from the fusion of secretory lysosomes with the cell surface, although additional work will be needed to test this hypothesis.

The differences in a3 localization between MCF10a and the breast tumor cell lines suggest that the intracellular localization patterns of a3 may be altered in breast cancer. As stated above, a3 localizes to late endosomes and lysosomes in osteoclast progenitor cells and to the plasma membrane in mature osteoclasts [7]. In pancreatic β cells, a3 localizes to insulin-containing secretory vesicles and may participate in insulin release as well as in acid-dependent insulin processing [15]. These studies, in addition to our findings, support the previously suggested concept that localization of subunit a isoforms depends upon the cellular context in addition to the expression level of the isoform [15].

The expression of the subunit a isoforms in human breast cancer had not previously been assessed prior to this study. Our results measuring a3 mRNA expression in the breast cancer cDNA array demonstrate a significant upregulation of a3 ranging from 2.5-47 fold in every breast cancer sample tested relative to normal tissue. In general, the highest degree of overexpression occurred in the higher-stage tumor samples. Our immunohistochemistry results demonstrate that a3 expression is greater in invasive breast carcinoma relative to adjacent ductal carcinoma *in situ* and relative to adjacent normal, hyperplastic, and solid tumor tissue. Overall, these results demonstrate for the first time that a3 expression is greatest in invasive breast cancer relative to noninvasive tumors and normal tissue.

The mechanisms by which a3-containing V-ATPases contribute to a more invasive or migratory phenotype in cancer cells are unclear. It has been hypothesized that plasma membrane V-ATPases contribute to cancer cell invasion by providing an acidic extracellular microenvironment that promotes the activity of pH-dependent proteases, such as the cathepsins, required for extracellular matrix degradation [1, 3, 26, 32, 46, 47]. Intracellularly, a3-containing V-ATPases may promote the trafficking of molecules that are important for invasion [48]. The contribution of V-ATPases to cell migration also remains poorly understood, although the ability of the pump to bind actin or to create a local increase in cytosolic pH that promotes actin polymerization may play important roles [1, 49, 50]. The pump may also promote the proper localization of molecules involved in migration, such as Rac1 and EGFR [24].

While a3 expression was generally greater in the cDNA samples of increased tumor stage, there was considerable variation in expression level. This suggests that some, though not all, tumors of a more advanced stage upregulate a3. It is interesting to note that a3 is also overexpressed in many noninvasive, stage I tumor samples, suggesting that a3 plays other roles in roles in cancer cells beyond promotion of metastasis. Indeed, the V-ATPase has previously been implicated in cancer cell survival, proliferation, and drug resistance [25, 30, 51–59] For example, cancer cells face a larger than normal acid burden as a result of their reliance on glycolytic metabolism. Plasma membrane V-ATPase activity allows for the removal of this acid from the cytosol, promoting cell survival [1]. a3 expression in early-stage tumors may also signal increased metastatic potential. Future studies are required to address these questions.

a3 expression has previously been detected on the plasma membrane of invasive pancreatic cancer cells [26] and was shown to be involved in the *in vitro* invasion and migration as well as *in vivo* metastasis of mouse melanoma cells [32]. a4 has previously been reported to be overexpressed in human glioma samples and to be involved in glioma cell invasion [60]. Previous work in our laboratory also demonstrated a role for a4 in MB231 cell invasion [22], although in the present study, a4 mRNA was upregulated in relatively few breast tumor samples. The a2 isoform was detected at the surface of ovarian cancer cells and was found to be moderately overexpressed in ovarian cancer tissues [28]. Interestingly, this study also reported a significant upregulation of a3 in several ovarian cancer cell lines, and immunohistochemistry demonstrated a significant upregulation of a3 in ovarian cancer relative to normal tissue [28]. Collectively, these studies suggest that a3 may play critical roles in cancers other than breast cancer, but that other a subunit isoforms may be important in a tumor-specific manner.

In summary, our results demonstrate for the first time that a3 is present at the leading edge of invasive breast cancer cells, is involved in breast cancer migration, and is overexpressed in invasive breast tumors. It has previously been shown that plasma membrane V-ATPase activity is critical for breast cancer cell invasion and migration *in vitro* [21]. The findings from the present study suggest that targeting a3-containing plasma membrane V-ATPases may represent a novel strategy for inhibiting breast cancer metastasis *in vivo*. This strategy would likely result in limited systemic side effects given the relatively restricted expression of a3-containing V-ATPases localized to the cell surface [3, 6]. While an a3-inhibitor would compromise osteoclast function, the fact that breast cancer cells recruit osteoclasts to invade bone suggests that such reduced osteoclast function would represent a beneficial side-effect of any drug targeting a3-containing V-ATPases. Future work will focus on the *in vivo* consequences of targeting a3-containing V-ATPases for breast cancer metastasis and assessing the mechanisms by which a3 contributes to breast cancer cell metastasis, survival, and proliferation.

## MATERIALS AND METHODS

### Materials and antibodies

DMEM, DMEM/F12, F12, Opti-MEM™, FBS, horse serum, penicillin-streptomycin, PBS, 0.05% trypsin-EDTA, the Alexa Fluor^®^ 488-conjugated goat anti-chicken secondary antibody, the Alexa Fluor^®^ 568 phalloidin antibody, the Alexa Fluor^®^ 594-conjugated goat anti-rabbit antibody, and ProLong^®^ Gold were purchased from Invitrogen. Aprotinin, leupeptin, and pepstatin were purchased from Roche Molecular Biochemicals. Pre-cast polyacrylamide mini-protean TGX gels, Tween 20, SDS, nitrocellulose membranes, and horseradish peroxidase-conjugated goat anti-mouse IgG were purchased from Bio-Rad. The chemiluminescence substrate for horseradish peroxidase was purchased from General Electric and the signal detected using Kodak BioMax Light film. The chicken monoclonal a3 antibody that recognizes the V-ATPase V_0_a3 subunit was described previously [36]. A mouse monoclonal antibody recognizing α-tubulin was purchased from Genscript. Rabbit polyclonal antibodies recognizing Giantin, EEA1, and LAMP2A and the rabbit monoclonal antibodies recognizing Rab7 and cortactin were obtained from Abcam. Concanamycin A was purchased from Bioviotica and epidermal growth factor was purchased from Peprotech. The siRNA pool against subunit a3, the Dharmafect 1 transfection reagent, and siRNA buffer were purchased from Dharmacon. Fluoroblok inserts with 8-μm pores were purchased from BD Biosciences. The breast cancer cDNA array was purchased from Origene and the Superfrost Plus microscope slides were purchased from Fisher Scientific. The Dako antibody diluent was purchased from Dako and the FITC-conjugated donkey-antichicken IgG secondary antibody was obtained from Jackson ImmunoResearch Laboratories. PMSF, calcein AM, streptavidin, and all other chemicals were purchased from Sigma.

### Cell culture

The human breast cancer cell line MDA-MB231 and the human breast epithelial cell line MCF10a were purchased from American Type Culture (ATCC). MCF10CA1a cells were provided by Dr. Yibin Kang (Princeton University) and SUM149 cells were provided by Dr. Charlotte Kuperwasser (Tufts University). All cells were grown in Falcon™ T-75 flasks in a 95% air, 5% CO_2_ humidified environment at 37°C. MB231 cells were grown in Dulbecco's Modified Eagle's Medium (DMEM) with phenol red, 25mM D-glucose, 4mM l-glutamine, and 1mM sodium pyruvate supplemented with 10% FBS, 60 μg/ml penicillin, and 125 μg/ml streptomycin. MCF10a and MCF10CA1a cells were cultured in DMEM/F12 medium containing 5% horse serum, 20 ng/ml epidermal growth factor, 0.5 μg/ml hydrocortisone, 100 ng/ml cholera toxin, 10 μg/ml insulin, 60 μg/ml penicillin, and 125 μg/ml streptomycin. SUM149 cells were cultured in F12 medium containing 5% FBS, 5 μg/ml insulin, 1 μg/ml hydrocortisone, 60 μg/ml penicillin, and 125 μg/ml streptomycin.

### Immunocytochemistry

Cells were plated on poly-d-lysine coated round coverslips in 24-well plates. For all immunofluorescence experiments, cells were fixed with 4% paraformaldehyde for 10 min, permeabilized with 0.1% Triton X-100 for 10 min, and then blocking was performed using 1% bovine serum albumin in PBS for 30 minutes. To assess the plasma membrane localization of subunit a3, cells were grown to confluence and a 200 μl pipette tip was used to scratch the confluent monolayer to create a wound to induce migration. Migrating cells and non-migrating control cells were then incubated in serum-free medium for an additional 4 h. After fixation, permeabilization, and blocking, cells were incubated with the anti-a3 antibody (1:400 dilution) overnight at 4°C. The cells were then rinsed with PBS and then incubated with the Alexa Fluor^®^ 488-conjugated goat anti-chicken secondary (1:500 dilution) and Alexa Fluor^®^ 568 phalloidin to stain F-actin (1:250 dilution). After 1 h of incubation at room temperature, the cells were washed with PBS. The cells were prepared for viewing using ProLong^®^ Gold mounting medium and allowed to cure at room temperature for 24 h. To quantify plasma membrane V-ATPase staining, 50-80 cells from three separate experiments were counted, and the percentage of cells showing plasma membrane a3 was determined. To determine whether a3 colocalizes with the leading edge marker cortactin during breast cancer cell migration, MCF10CA1a, MB231, and SUM149 cells were grown to confluence and a wound induced in the cell monolayer as described above. After fixation, permeabilization and blocking, cells were incubated overnight at 4°C with the anti-a3 antibody (1:400) and the anti-cortactin antibody (1:1000) followed by incubation with the Alexa Fluor^®^ 488-conjugated goat anti-chicken secondary (1:500 dilution) and the Alexa Fluor^®^ 594-conjugated goat anti-rabbit secondary (1:500 dilution) for 1 h at room temperature. Mounting, imaging, and analysis were performed as described above. To assess colocalization of subunit a3 with various intracellular markers, cells were plated slightly sub-confluently and then fixation, permeabilization, and blocking performed as described above. The cells were incubated overnight at 4°C with the anti-a3 antibody (1:400) and the anti-Giantin antibody (to stain Golgi, 1:1000 dilution), the anti-LAMP2A antibody (to stain lysosomes, 1:500 dilution), the anti-EEA1 antibody (to stain early endosomes, 1:100 dilution), or the anti-Rab7 antibody (to stain late endosomes, 1:150 dilution). Next, the cells were incubated with the Alexa Fluor^®^ 488-conjugated goat anti-chicken secondary (1:500 dilution) and the Alexa Fluor^®^ 594-conjugated goat anti-rabbit secondary (1:500 dilution) for 1 h at room temperature. Mounting and imaging were performed as described above. All images were obtained using a Leica TCS SPE confocal microscope. For determination of a3 plasma membrane localization and colocalization with cortactin, images were taken with a 40x objective. For determination of a3 localization to intracellular compartments, images were taken with a 63x objective.

### RNA interference

siRNA experiments were performed as described previously [23]. Briefly, an siRNA pool specific for the subunit a3 isoform of the V-ATPase was purchased from Dharmacon. The pool contains four siRNAs specific for the a3 subunit isoform. MB231 cells were plated in wells of a six-well plate at 3.5 × 10^5^ cells/dish and incubated overnight. Cells were transfected with 100 nM siRNA directed against a3 or 25 nM scrambled control siRNA according to the directions of the manufacturer. Briefly, the siRNA was diluted in Opti-MEM™, allowed to incubate for 5 min, and then mixed with Dharmafect 1 transfection reagent. The siRNA/transfection reagent mixture was incubated for 20 min at room temperature and then added to the appropriate volume of Opti-MEM™, and 2 mL was added to each well. After incubation of cells with siRNA for 24 h, the media were changed to DMEM/10% FBS and cells were incubated for an additional 48 h. Cell lysis, *in vitro* migration assays, or treatment with concanamycin A were performed 72 h post-transfection. The viability of control and a3-siRNA transfected cells treated with or without 100 nM concanamycin A for 24 h was determined using a trypan blue exclusion assay according to the manufacturer's instructions.

### Cell lysis and western blotting

Cell monolayers were washed twice in cold PBS and then collected by scraping into 100 μl lysis buffer (150 mM NaCl, 1% Triton-X-100, 50 mM Tris-HCl pH 8.0, 1 mM PMSF, 2 μg/ml aprotinin, 5 μg/ml leupeptin, and 1 μg/ml pepstatin. Cell suspensions were agitated for 30 minutes at 4°C to allow lysis and then centrifuged at 16,100 x *g* for 20 minutes to clear unbroken cells and nuclei. The protein concentration of the supernatant was then determined by the Lowry method [61]. SDS sample buffer was added to the lysates and proteins were separated by SDS-PAGE on 4-15% gradient acrylamide gels. The presence of subunit a3 and tubulin were detected by overnight incubation at 4°C using chicken and mouse monoclonal antibodies, respectively, followed by incubation with a horseradish peroxidase-conjugated secondary antibody at room temperature for 1 h. Blots were developed using chemiluminescent detection reagents. Western blots were quantified using ImageJ software with corrections made for loading.

### Migration assays

Assays for *in vitro* migration were performed as described previously [21]. Fluorblok inserts with an 8-um pore size membrane were placed into the wells of a 24-well plate [62]. Cells were harvested by trypsinization and brought to a concentration of 1.0 × 10^5^ cells/ml in DMEM plus phenol red, 25 mM D-glucose, 4 mM l-glutamine, and 1 mM sodium pyruvate (termed ‘Medium’) containing DMSO or 100 nM of the V-ATPase-specific inhibitor concanamycin A. Cells were incubated for 15 min on at 37°C, and then 5.0 × 10^4^ cells were seeded onto the membranes of the inserts. Inserts were then placed into wells of a 24-well plate that contained 500 ul of Medium plus 5% FBS to serve as a chemoattractant. After an average incubation of 20 hours at 37°C, membranes were placed into wells containing 4 μg/ml calcein AM in PBS and incubated for 5 min at 37°C in 5% CO_2_. Cells that had migrated to the *trans*-membrane were imaged using a Zeiss Axiovert 10 fluorescence microscope, with an average of 10 images taken per well. The number of migrating cells was averaged over three wells per condition with all experiments performed at least three times.

### qPCR

The breast cancer cDNA array II was obtained from Origene. The cDNA array contained cDNA from 5 normal breast tissues and 43 breast tumor tissues of varying stage and receptor status. qPCR was performed using primers for beta-actin (supplied by Origene), human subunit a3 (forward: 5′-GACCGACAGGAGGAAAACAAG-3′, reverse: 5′-GT AGGAGGCGGTGTTGGA-3′), or human subunit a4 (forward: 5′- GGAAATCCCAGCTGCAGGC-3′, reverse: 5′- GCAGCCCAGGCAGTACTCG-3′) according to the manufacturer's instructions using SYBR Green qPCR master mix. The PCR cycling sequence consisted of an activation step of 50°C for 2 min followed by 40 cycles of 30 s at 95°C, 1 min at 55°C, and 1 min at 72°C to allow for denaturation, annealing, and extension, respectively. To assess a3 expression relative to expression in the first normal tissue, the ΔΔC_t_ method was used for analysis using beta-actin as the reference gene. Data were presented as the average relative expression over two experiments.

### Immunohistochemistry

Formalin fixed, paraffin-embedded tumor blocks, obtained from the pathology research center at Massachusetts General Hospital, were cut at 5 μm thickness. Sections were placed onto Superfrost Plus microscope slides and stored at room temperature until use. Sections were hydrated in PBS for 10 min. After deparaffinization in xylene (100% two times for 5 min), and dehydration in a graded ethanol series (100%, 90%, 75%, 50%, 30%, each for 5 min), sections were washed 3 times for 5 min in PBS. Sections were either immediately blocked with 1% bovine serum albumin for 15 min prior to antibody incubation or counterstained. Counterstaining was performed with hematoxylin and eosin (H&E) to help visualize tissue morphology. H&E stained sections were scanned using a NanoZoomer 2.0-RS (Hamamatsu). After blocking, sections were incubated with the a3 antibody chicken monoclonal antibody generated against the a3 subunit isoform of the V-ATPase, diluted in Dako antibody diluent (1: 100), and incubated overnight at 4°C in a humidity chamber. The fluorophore conjugated secondary antibody used in this study (FITC-conjugated donkey anti-chicken IgG) was diluted in Dako antibody diluent (1: 100) and slides were incubated for 1 h prior to 3 washes for 5 min in PBS. Confocal images were acquired on a Nikon A1R confocal microscope with NIS Elements (Nikon Instruments).

## SUPPLEMENTARY FIGURES


