# Supplementary material for: The a3 isoform of subunit a of the vacuolar ATPase localizes to the plasma membrane of invasive breast tumor cells and is overexpressed in human breast cancer
Source: Oncotarget. 2016 Jun 15;7(29):46142–57. doi: 10.18632/oncotarget.10063 (PMC5216787; doi:10.18632/oncotarget.10063)
Supplement: Supplementary file 1 [file oncotarget-07-46142-s001.pdf]

The a3 isoform of subunit a of the vacuolar ATPase localizes to the plasma membrane of invasive breast tumor cells and is overexpressed in human breast cancer

SUPPLEMENTARY FIGURE

A.

	Human a1	Human a2	Human a3	Human a4
**Mouse a1**	**95.91**	54.05	48.03	62.65
**Mouse a2**	53.73	**91.59**	52.68	52.4
**Mouse a3**	48.06	52.98	**84.44**	48.42
**Mouse a4**	61.96	52.78	47.65	**86.07**

B.

```
Mouse a3    651 GTPLYLLRQHRHRRNTQRRPAGQQDEDTDKLLASPDASTLENSWSPDEEK    700
                ||||:||  |||||..:||||.:|:|:...||..||||.  |.||.||||
Human a3    650 GTPLHLL--HRHRRRLRRRPADRQEENKAGLLDLPDASV--NGWSSDEEK    695

Mouse a3    701 AGSPGD-EETEFVPSEIFMHQAIHTIEFCLGCISNTASYLRLWALSLAHA    749
                ||...| ||.|.||||:.|||||||||||||||:||||||||||||||||
Human a3    696 AGGLDDEEEAELVPSEVLMHQAIHTIEFCLGCVSNTASYLRLWALSLAHA    745
```

C.

```
a1      FGYLVILIFYKWTAYDAHTSENAPSLLIHFINMFLFSYPESGYSMLYSGQKGIQCFLVVV      642
a2      FGYLIFMIFYKWLVFSAETSRVAPSILIEFINMFLFP--ASKTSGLYTGQEYVQRVLLVV      655
a3      FGYLVFLVIYKWLCVWAARAASAPSILIHFINMFLFSHSP-SNRLLYPRQEVVQATLVVL      639
a4      FGYLVFMIIFKWCCFDVHVSQHAPSILIHFINMFLFNYSDSSNAPLYKHQQEVQSFFVVM      646
        ****::::::**    .  :  ***:**.*******         **  *: :*  ::*:

a1      ALLCVPWMLLFKPLVLRRQYLRRKHLGTLNFGGIRVGNGPTEE-----DAEI-IQHDQLS      696
a2      TALSVPVLFLGKPLFLLWLHNGRSCFGVNRSGYTLIRKDSEEEVSLLGSQDI----EEGN      711
a3      ALAMVPILLLGTPLHLLHRHRRRLRRRPA--------DRQEENKAGLLDLPDASVNGWSS      691
a4      ALISVPWMLLIKPFILRASHRKSQLQA------SRIQEDATENIEGDSSSPS-SRSGQRT      699
        :   ** ::* .*: *   :                 .   *:     .          .

a1      T-HSEDADEPSEDEVFDFGDTMVHQAIHTIEYCLGCISNTASYLRLWALSLAHAQLSEVL      755
a2      H-QVEDGCREMACEEFNFGEILMTQVIHSIEYCLGCISNTASYLRLWALSLAHAQLSDVL      770
a3      DEEKAGGLDDEEEAELVPSEVLMHQAIHTIEFCLGCVSNTASYLRLWALSLAHAQLSEVL      751
a4      S-ADTHGALDDHGEEFNFGDVFVHQAIHTIEYCLGCISNTASYLRLWALSLAHAQLSEVL      758
              .        :  .: :: *.**:**:****:********************:**
```

Supplementary Figure S1: Sequence comparison of human and mouse V-ATPase a subunit isoforms. A. Percent identity between the mouse and human protein sequences for subunit a isoforms a1-a4 were determined using Clustal Omega multi-sequence alignment [37–39]. **B.** Mouse and human a3 protein sequences were aligned using the EMBOSS Water pairwise alignment [39]. Region displayed contains the mouse sequence against which the chicken monolconal antibody was prepared (highlighted in grey). The region highlighted in yellow indicates the corresponding human sequence. *Straight lines* (|) indicate identical residues. *Colons* (:) indicate strongly conserved residues. *Periods* (.) indicate weakly conserved residues [37–39]. **C.** Human protein sequences for a1-a4 were aligned using Clustal Omega multi-sequence alignment. The region highlighted in yellow indicates the corresponding human sequence to which the antibody was prepared. *Asterisks* (*) indicate identical residues. *Colons* (:) indicate strongly conserved residues. *Periods* (.) indicate weakly conserved residues [37–39].
